# A pan-cancer analysis of matrisome proteins reveals CTHRC1 and a related network as major ECM regulators across cancers

**DOI:** 10.1371/journal.pone.0270063

**Published:** 2022-10-03

**Authors:** Keerthi Harikrishnan, Srinivas Sheshagiri Prabhu, Nagaraj Balasubramanian

**Affiliations:** Indian Institute of Science Education and Research (IISER) Pune, Pashan, Pune, Maharashtra, India; University of Patras, GREECE

## Abstract

The extracellular matrix in the tumour microenvironment can regulate cancer cell growth and progression. A pan-cancer analysis of TCGA data from 30 cancer types, identified the top 5% of matrisome genes with amplifications or deletions in their copy number, that affect their expression and cancer survival. A similar analysis of matrisome genes in individual cancers identified CTHRC1 to be significantly altered. CTHRC1, a regulator of collagen synthesis, was identified as the most prominently upregulated matrisome gene of interest across cancers. Differential gene expression analysis identified 19 genes whose expression is increased with CTHRC1. STRING analysis of these genes classified them as ‘extracellular’, involved most prominently in ECM organization and cell adhesion. KEGG analysis showed their involvement in ECM-receptor and growth factor signalling. Cytohubba analysis of these genes revealed 13 hub genes, of which MMP13, POSTN, SFRP4, ADAMTS16 and FNDC1 were significantly altered in their expression with CTHRC1 and seen to affect survival across cancers. This could in part be mediated by their overlapping roles in regulating ECM (collagen or fibronectin) expression and organisation. In breast cancer tumour samples CTHRC1 protein levels are significantly upregulated with POSTN and MMP13, further supporting the need to evaluate their crosstalk in cancers.

## Introduction

Extracellular matrix (ECM) is a dynamic interconnected mesh of macromolecules that provides structural support and also regulates cellular behaviour via mechanical and biochemical cues. It regulates several cellular processes including proliferation, differentiation, migration, invasion and survival [1]. ECM composition is tightly regulated by the cells and changes in ECM production, secretion, deposition and remodelling are reported in pathological diseases such as atherosclerosis, fibrosis, skeletal disorders, vascular disorders and cancer [2, 3]. ECM composition also varies between the tumour cells, tumour stroma and it is distinctly different across the metastatic sites [4, 5]. Recent studies characterizing changes in the composition of ECM in normal and tumour microenvironments have emphasized the importance of ECM and its contribution towards developing novel biomarkers and therapeutic targets [5–7].

The “Matrisome” pioneered by *Naba et al*., is an ensemble of genes that codes for core ECM proteins, ECM associated growth factors, ECM regulators and other ECM associated factors [6, 8]. It accounts for 4% of the human and mouse genome and reflects the composition of proteins in normal and tumour tissues [3, 9]. Since the publication of MatrisomeDB in 2012, the understanding of the role of ECM in cancers has significantly enhanced. Matrisome proteins like Insulin like growth factor binding protein 3, 4, 5 (IGFBP3, IGFBP4, IGFBP5), Cellular Communication Network factor (CCN) family, Thrombospondin2 (THBS2), Tenascin N (TNN) and Von Willebrand factor A 9 (VWA9) are detected primarily in cancer tissues [10] while Lysyl oxidase like 2 (LOXL2) [11], Cartilage oligomeric matrix protein (COMP), Periostin (POSTN) [12], Tenascin N (TNC) [13], Tenascin X (TNX) [13] and Fibronectin (FN) (EIIIA and EIIIB variant) [14] have been reported to be upregulated in cancers. Oncomine analysis of core matrisome genes in the lung, gastric, ovarian and colon cancers show that a signature of 9 genes Collagen type XI alpha 1 chain (COL11A1), Secreted phosphoprotein 1 (SPP1), Microfibrillar associated protein 2 (MFAP2), Collagen type X alpha 1 chain (COL10A1), Biglycan (BGN), Cartilage oligomeric matrix protein (COMP), Agrin (AGRN) and Matrix remodelling associated 5 (MXRA5) is associated with poor survival and is involved in regulating cancer hallmarks such as epithelial to mesenchymal transition (EMT), and angiogenesis [15]. Data mining of 10 NSCLC microarray datasets has identified 29 ECM signature genes which were found to be consistently upregulated in patients with NSCLC and also predicts prognosis [16]. Analysis of 12 cancer types (lung, pancreas, prostate, kidney, stomach, colon, ovary, breast, liver, bladder and skin) shows that tumour matrisome index (TMI) is associated with disease progression and poor clinical outcome [17]. In addition, tumours with high TMI show enrichment for Mage family member A3 (MAGEA3) and CD8 positive T cells and also display high expression of B7-H3 which is negatively associated with clinical outcome in solid tumours [18].

Pan-Cancer analysis of transforming growth factor ß (TGFß) associated ECM gene expression shows a set of matrisome genes to be upregulated in cancer and the expression is associated with a worse prognosis. This study also reveals an association of aberrant ECM expression with immunosuppression in cancers [17, 19]. Cell-cell adhesion, Forkhead box O (FOXO), Wnt pathways are found to control matrisome in most cancer types whereas tumour protein 53 (TP53), Notch and TGFß signalling pathways regulate matrisome genes in some cancers [20].

Using a multi-omics approach and machine learning, several landmark matrisome genes have been identified from 74 clinical and molecular subtypes of cancers that show prognostic significance [21]. Bioinformatic analysis of the copy number alterations (CNA’s) reveals that matrisome genes display a disproportionately high number of CNA’s and mutations compared to the rest of the genome [22] across cancers. This increase in the genome alterations of matrisome was further predictive of prognosis across cancer types. Together, these findings highlight a significant role of ECM genes in cancer progression.

While recent studies have evaluated the role of matrix protein families or individual cancer matrisome signatures, our study was aimed at identifying matrisome genes that can act as vital ECM regulators in pan-cancer analysis and individual cancers. Our pan-cancer analysis of matrisome genes for copy number variation (amplification or deletion), relative expression and effect on cancer survival identified collagen triple helix repeat containing 1 (CTHRC1) as a major pan-cancer ECM regulator. Its role is further supported by CTHRC1 being identified as the most prominently regulated ECM protein across individual cancers. Further, network analysis reveals CTHRC1 could work with matrisome genes Periostin (POSTN), Matrix metalloproteinase 13 (MMP13), Secreted frizzled related protein 4 (SFRP4), Fibronectin type III domain containing 1 (FNDC1) and ADAM metallopeptidase with thrombospondin type 1 motif (ADAMTS16) in regulating the impact ECM has on cancers.

## Materials and methods

### Data sources

The list of matrisome genes was downloaded from the matrisome database [23]. TCGA Pan-Cancer copy number data calculated using the GISTIC2 threshold method was downloaded from the UCSC Xena browser [24]. TCGA and GTEx data was used to perform the expression, survival, correlation, cooccurrence disease stage and protein analysis. The analysis was restricted to 30 types of cancers from TCGA which include: Adrenocortical Carcinoma (ACC), Bladder Urothelial Carcinoma (BLCA), Breast Invasive Carcinoma (BRCA), Cervial Squamous Cell Carcinoma (CESC), Cholangiocarcinoma (CHOL), Colon Adenocarcinoma (COAD), Lymphoid Neoplasm Diffuse Large B-cell Lymphoma (DLBC), Esophageal Carcinoma (ESCA), Glioblastoma Multiforme (GBM), Head and Neck Squamous Cell Carcinoma (HNSC), Kidney Chromophobe (KICH), Kidney Renal Clear Cell Carcinoma (KIRC), Kidney Renal Papillary Cell Carcinoma (KIRP), Brain Lower Grade Glioma (LGG), Liver Hepatocellular Carcinoma (LIHC), Lung Adenocarcinoma (LUAD), Lung Squamous Cell Carcinoma (LUSC), Ovarian Serous Cystadenocarcinoma (OV), Pancreatic Adenocarcioma (PAAD), Pheochromocytoma and Paraganglioma (PCPG), Prostate Adenocarcinoma (PRAD), Rectum Adenocarcinoma (READ), Sarcoma (SARC), Skin Cutaneous Melanoma (SKCM), Stomach Adenocarcinoma (STAD), Testicular Germ Cell Tumours (TGCT), Thyroid Carcinoma (THCA), Thymoma (THYM), and Uterine Corpus Endometrial Carcinoma (UCEC), Uterine Carcinosarcoma (UCS).

### Copy number variation analysis

First genes were clustered based on their functionality (ECM genes, Proteoglycans etc.). These gene clusters were prepared as an excel file containing a single column with gene symbols as cell entries and this list represented the genes of interest whose copy number variations were to be analyzed. There were 10845 samples in total and the gene-level copy number estimate values of -2, -1, 0, 1, 2 represented deep deletion, shallow deletion, no change, amplification and gain respectively. The excel file retrieved after extracting the dataset was in the genomic Matrix format (ROWs (identifiers) x COLUMNs (samples)). Next, we wrote a code in Python to find the gene-level copy number estimate values of our genes of interest across all 10845 samples. To achieve this, we had to explore our list in the database retrieve the corresponding values for the samples and process the data to calculate the number of deep deletions and gain. The top 5% of the genes (n = 104) with amplification or deep deletion were then used for further analysis. The code used to perform the analysis can be made available upon written request.

### Expression, survival, correlation and disease stage analysis

RNA expression for the matrisome genes was analyzed using the GEPIA2 portal [25] which contains the expression data for 9736 tumour samples and 8587 normal samples and the data is processed using a standard processing pipeline. The data from 30 cancer types are represented in a box plot (log scale) as mean ± standard deviation (S.D). We also evaluated the expression of the matrisome genes by pathological stage using the stage plot function in the database. The data from 30 cancer types are represented in a violin plot (log scale) which shows the distribution of data. p-value less than 0.05 was considered statistically significant. Survival analysis for all the matrisome genes was performed using TCGA data through the GEPIA2 portal. A custom cutoff was set for the survival analysis with the top 75 percentile being classified as “high” and the bottom 25 percentile classified as “low”. This classification was used to evaluate the significance of the difference in effect on survival that exists between the “high” and “low” groups. Log-rank p values and the hazard ratio (HR) with 95% confidence interval were calculated using the GEPIA2 web portal and a p-value of less than 0.05 was considered statistically significant. This was used to classify individual cancers as affecting survival (p<0.05) or not (p> 0.05).

Percentage effect on expression and survival was calculated as follows:

$$\text{Percentage}\;\text{affected}=\frac{\text{number}\;\text{of}\;\text{cancers}\;\text{significantly}\;\text{affected}}{\text{total}\;\text{number}\;\text{of}\;\text{cancers}\left(\text{n}=30\right)}$$

**Fig 1:**
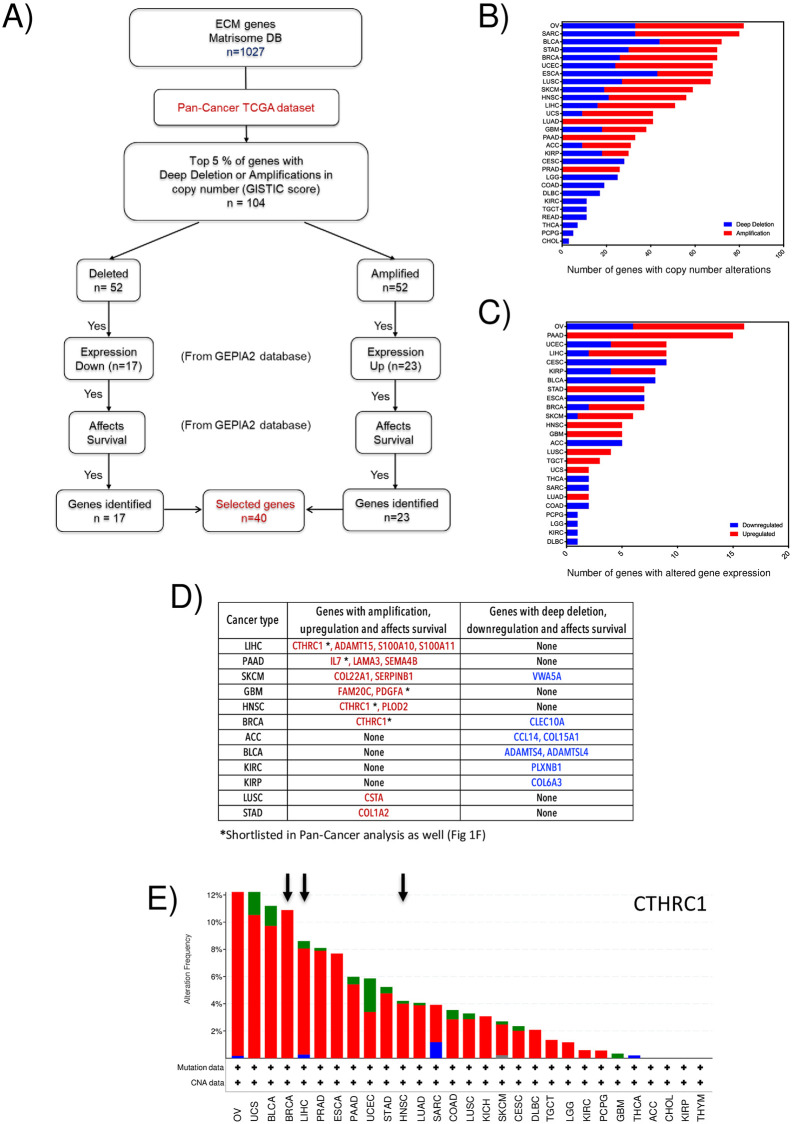
Cancer type-specific analysis of matrisome genes. **A)** Schematic shows the steps involved in shortlisting genes from the individual TCGA cancer dataset based on copy number (GISTIC score), expression and survival (GEPIA2 database). The number of genes that met the criteria for each stage and were eventually shortlisted are indicated in the box for genes identified. Their occurrence in two or more cancers was used for the final selection (n = 1). **B**) Based on their copy number, the number of deep deleted (BLUE bar) or amplified (RED bar) matrisome genes in individual cancers (n = 28) were arranged in the descending order based on total genes affected (BLUE+RED). **C**) Based on their mRNA expression, the top 5% of matrisome genes in individual cancers (n = 28) that are downregulated (BLUE bar) or upregulated (RED bar) were arranged in descending order based on total genes affected (BLUE+RED). **D**)Table lists the individual cancers with one or more amplified and upregulated (RED) or deleted and downregulated (BLUE) genes that affect survival. Genes marked with an asterisk (*) are also shortlisted in the pan-cancer analysis (Fig 2F). Genes marked in **bold** are affected by more than one cancer type. **E**) Graph represents the mutational (GREEN), copy number amplification (RED) and deletion (BLUE) analysis of CTHRC1 in 30 individual cancers. Arrows point to the individual cancers where CTHRC1 is also selected as detailed above (***Fig 1D***).

Univariate and Multivariate Survival analysis for Figs 1B and 3B was performed using TCGA data with the survival analysis code in the TCGA2STAT package in R (version 3.6.3)Analysis 2 (univariate), 3 (multivariate) [26].

Correlation analysis for CTHRC1 along with its 10 hub genes across 30 cancer types was done using the Spearman correlation coefficient. Correlation where p < 0.05 was considered statistically significant.

### Scoring of genes based on expression and effect on survival

**Fig 2:**
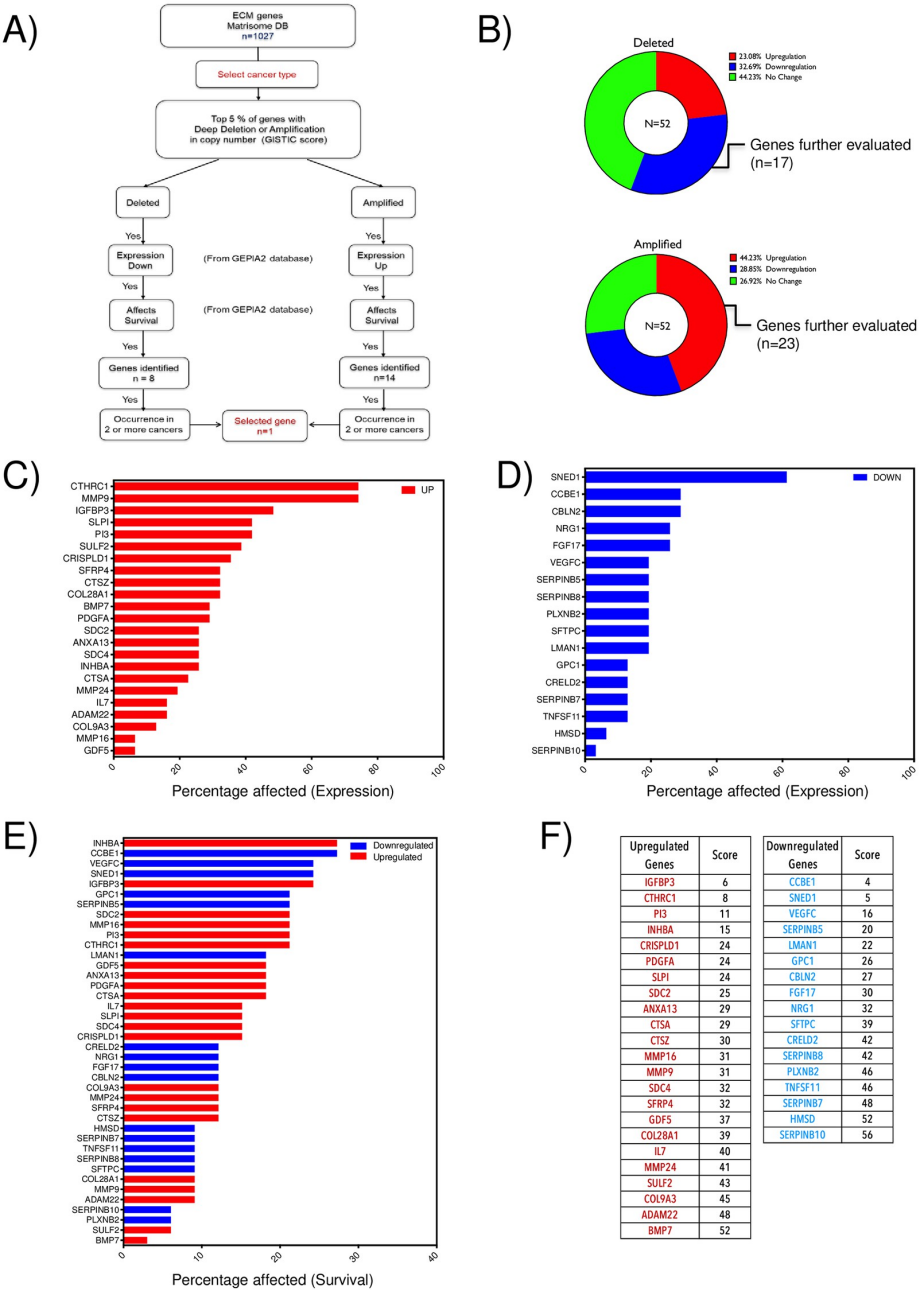
Pan-cancer analysis of matrisome genes. **A)** Schematic shows the steps involved in shortlisting genes from the pan-cancer TCGA dataset based on copy number (GISTIC score), expression and survival (GEPIA2 database). The number of genes shortlisted at each stage of the selection process is indicated in each box. **B**) Deleted (n = 52) and amplified (n = 52) genes were classified based on their mRNA expression as downregulated (BLUE), upregulated (RED) and no change (GREEN). The nested bar graph represents the percentage of each for deleted (top graph) and amplified (bottom graph) genes. **C)** Bar graph shows the percentage of cancers where the top 5% of amplified matrisome genes are also upregulated (n = 23). Genes are arranged in descending order (RED–represents upregulated genes). **D)** Bar graph shows the percentage of cancers where the top 5% of deleted matrisome genes are also downregulated (n = 17). Genes are arranged in descending order (BLUE–represents downregulated genes). **E)** Bar graph shows the percentage of survival in cancers for the top 5% of matrisome genes shortlisted in **C** (RED bar) and **D** (BLUE bar). These are arranged in descending order. **F)** Tables list genes in the ascending order of their score calculated based on their position in the expression (***C*, *D***) and survival (***E***) graphs (as detailed in methods). A lower score is indicative of a higher position in these graphs. Upregulated genes are listed in RED and downregulated genes are listed in BLUE.

Genes selected based on their copy number agreeing with mRNA expression (Fig 2A) were arranged in descending order based on their upregulation or downregulation across cancers. The data plotted in these graphs represent percentage alteration calculated as above. A score was assigned for each gene based on their position in the upregulated or downregulated graphs respectively. The top gene was assigned a score of 1 and this score increased by 1 point for the next gene. This was done for all genes in the graph.

All of the above genes (upregulated or downregulated) were further arranged based on their effect on survival across cancers in descending order. A score was assigned for each gene based on its position in the survival graph. The top gene was assigned a score of 1 and this score increased by 1 point for the next gene. This was done for all genes in the graph.

These two scores were added to obtain the final score for upregulated and downregulated genes. They were arranged in a table based on their score (low to high). A low score indicates a higher ranking in expression (up or down) and survival.

### Mutation and co-occurrence analysis

Mutation analysis for 8 shortlisted genes (based on expression, survival data as mentioned above) was analyzed using the cBioPortal database across 30 cancer types. It showed the mutational burden along with the copy number analysis in the patient samples. We also used this portal to identify the co-expressed genes of CTHRC1(30 cancer types) in the cBioPortal using the cooccurrence-mutual exclusivity tab in the database. p less than or equal to 0.05 was considered statistically significant.

### Differential gene expression analysis

First, the data was preprocessed locally in R using the preprocessing script. The differential gene expression (DEG) analysis was performed (30 cancer types) in groups with high expression (75%) or low (25%) expression. Thresholds for DEG’s was set at fold change > 2, -2 with a p-value of 0.05. The following source code from the GitHub repository was used to perform the preprocessing and the DEG analysis [27–29]

### Venn diagram analysis for overlapping genes

Omics Box software and the Venn diagram tool [30] were used to identify the overlapping differentially expressed genes across cancer types. 20 genes that overlapped in 3 or more cancers were used for further analysis. Venn diagram tool was also used to identify the top hub genes that overlapped in survival, disease stage expression, correlation and co-occurrence analysis. 3 genes identified were then used to perform CPTAC analysis.

### Protein-protein interaction and functional enrichment analysis

STRING database [31] was used to identify the protein-protein interactions of CTHRC1 along with the differentially expressed genes across different cancer types. The list of proteins along with CTHRC1 was entered in the online database and a medium confidence interaction score of 0.4 was used to generate the full string network. The network was then exported as a high-resolution image. Functional enrichment analysis performed using the STRING database included biological process, molecular function, cellular compartment and KEGG pathways. FDR of < 0.05 was used to identify the gene ontology (GO) terms that were statistically significant.

### CytoHubba analysis

The PPI network constructed using the STRING database was sent to Cytoscape using the web link. Cytoscape (version 3.8.2) is an open-source software used for the visualization and analysis of protein-protein interaction networks. Using the cytoHubba tool in the software, we identified the top 14 hub genes based on the degree of connectivity. These hub genes were then used for further analysis.

### GeneMANIA PPI network analysis

GeneMANIA [32] was used to construct a PPI network of CTHRC1 along with its hub genes (POSTN, MMP13, FNDC1, SFRP4 and ADAMTS16) to identify if these genes are functionally related and discover genes that could be part of this functional network. The networks were generated using the following weighing methods: 1) Based on query genes and 2) Gene Ontology. In the gene ontology-based weighing method, Biological Process and Cellular component methods were used for building networks. GeneMANIA will build networks showing genetic interactions, protein-protein interactions, protein-DNA interactions, protein expression, similarity in protein domains, pathways and phenotypic screening profiles using the publicly available datasets.

### UALCAN analysis

UALCAN is a web-based tool for the analysis of CPTAC data from the TCGA cancer types. We used the UALCAN database [33] to analyze the protein levels of CTHRC1, POSTN, MMP13, SFRP4 and FNDC1 in breast cancer. Data were represented as mean ± S.D and a p-value of less than 0.05 were considered statistically significant.

## Results

### Pan-cancer analysis of altered matrisome genes

In this study, we first obtained the list of matrisome genes (n = 1027) from the Matrisome database (DB). We then analyzed the copy number variation of these genes using the Pan-Cancer TCGA dataset from the UCSC cancer genome browser (Fig 2A). Overall, there were 10845 samples in this dataset which were scored based on their copy number variations (GISTIC score from UCSC Xena browser) (Fig 2A). The top 5% of genes with deep deletions or amplifications were identified and shortlisted (104 genes). Using the GEPIA2 portal, we evaluated the mRNA expression of these 104 genes across 30 cancer types. Of these deep deleted and amplified genes, 32.69% showed reduced mRNA expression (17 genes) and 44.23% showed increased mRNA expression (23 genes) respectively (Fig 2B). Upregulated (Red Graph) and downregulated (Blue Graph) genes were arranged in descending order as per their change in pan-cancer expression (Fig 2C and 2D). Similarly, the effect of these genes on pan-cancer survival was determined using the GEPIA2 portal and genes were arranged in descending order (Fig 2E) (the colour code used for expression of genes was retained in the graph). The relative position of genes in the expression and survival graphs was used to score them as detailed in the methods section. The final score thus obtained was used to arrange upregulated (red) and downregulated (blue) genes in the descending order (Fig 2F). A low score indicates a higher ranking for the gene in expression and survival.

### Individual cancer analysis of altered matrisome genes

Further, we analyzed the copy number, expression and effect on survival of matrisome genes in individual cancers (Fig 1A). The pooling of cancer types in the above pan-cancer analysis could be sensitive to tumours with significant copy number alterations that could skew the selection of gene(s) of interest. Combining the pan- and individual cancer analysis could be better at identifying matrisome gene(s) of consequence. TCGA data for 30 individual cancer types obtained from the UCSC cancer genome browser was used for copy number analysis. This showed THYM and KICH cancers to lack deep deletions or amplifications in any of the matrisome genes (*data not shown*). Of the 28 remaining cancers, 15 showed both deep deletions and amplifications in their matrisome genes, while 10 showed only deep deletions and 3 had only amplifications. The total number of genes with amplification (red bar) and deep deletions (blue bar) for individual cancers were represented in the graph and arranged in descending order (Fig 1B). In each of these 28 cancers, we selected the top 5% of genes whose copy number is altered (either deleted or amplified) (Fig 1A). Using the GEPIA2 portal, we evaluated the mRNA expression of these genes. Genes with deep deletion and reduced mRNA expression or amplification and increased mRNA expression were shortlisted for each cancer (Fig 1A). The number of so-identified upregulated (red bar) and downregulated (blue bar) genes for each cancer were plotted and they were arranged in descending order (Fig 1C). We further tested if these shortlisted genes in their respective cancers significantly affected survival (Fig 1A). This revealed 14 amplified (red) and 8 deleted (blue) genes to affect survival in 12 individual cancers, listed in the table for Fig 1D. Of these genes, CTHRC1, PDGFA, and IL7 were also shortlisted as matrisome genes of interest in the pan-cancer analysis (Figs 1D and 2F). CTHRC1 was however the only gene that was affected in more than one individual cancer type (BRCA, HNSC and LIHC) (Fig 1D). Copy number variation and mutation analysis for CTHRC1 across cancers find it to be prominently amplified in most cancers (Fig 1E) including BRCA, HNSC and LIHC (marked by arrow). This led us to choose CTHRC1 as the matrisome gene of interest for further evaluation.

### Detailed Pan-cancer analysis of CTHRC1

**Fig 3:**
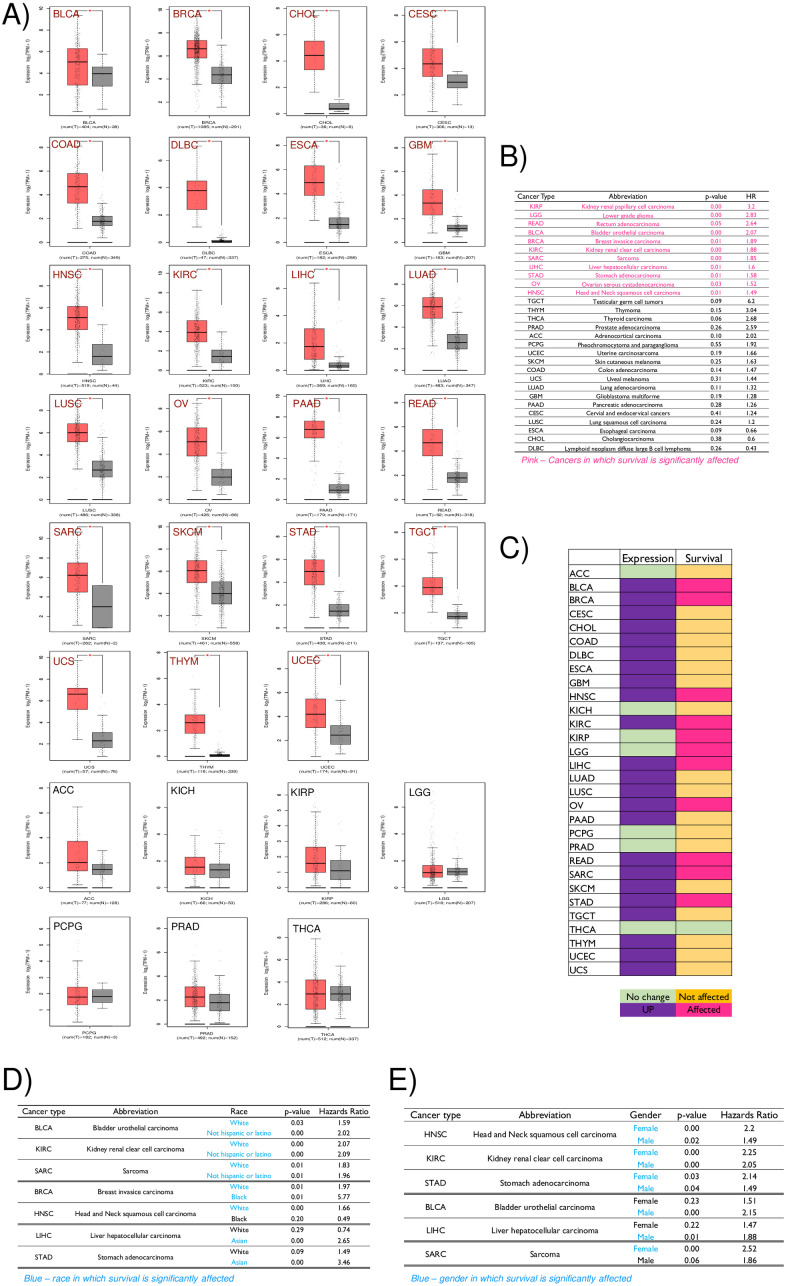
Pan-cancer analysis of CTHRC1 expression and its effect on survival. **A)** Graphs represent CTHRC1 expression data from the GEPIA2 portal in 30 different tumour types (T–RED bar) relative to normal (N–GREY bar). Cancers showing significant upregulation in CTHRC1 are listed first and labelled in RED. Those showing no significant change in expression are listed later and labelled in BLACK. Expression data are represented as mean ± standard deviation (S.D) on a log scale using a box plot. p-value of < 0.05 was determined as statistically significant. **B)** Table lists the results of univariate analysis of CTHRC1 expression on survival across 30 individual cancers. It shows the significance values (p-value) for survival in patients with “high” vs “low” CTHRC1 expression and their hazards ratio (HR). Cancers with significance p ≤ 0.05 are listed in PINK and p > 0.05 in BLACK in the descending order of their respective hazards ratio. **C)** Table shows CTHRC1 expression and survival data in 30 individual cancers. Upregulated (PURPLE), and comparable (GREEN) expression marked accordingly. Significant effect seen on survival is marked in PINK and lack thereof marked in ORANGE. **D-E**) Tables shows the multivariate survival analysis for CTHRC1 expression in the context of **(D)** race and **(E)** gender in selected cancers for which data is available. It shows the significance values (p-value) for survival in patients with “high” vs “low” CTHRC1 expression and their hazards ratio (HR) for comparison. Cancers with significance p < 0.05 are listed in their descending order of significance.

CTHRC1 (Collagen triple helix repeat containing-1) expression (evaluated in 30 individual cancers using GEPIA2 portal confirmed its overexpression in 23 cancers (Fig 3A—labels in red and Fig 3C–boxes coloured in purple). In 7 cancers no change in expression was observed (Fig 3A—labels in black and Fig 3C–boxes coloured in green). We also compared the effect CTHRC1 has on survival in 30 cancers using univariate and multivariate analysis. Using TCGA data univariate analysis was done to calculate the hazards ratio (HR), which captures the likelihood of CTHRC1 expression affecting survival in cancers (Fig 3B). The calculated significance showed CTHRC1 expression to significantly affect survival in 11 cancers (Fig 3B and 3C –boxes in pink). A comparison of the expression and survival data to identify cancers significantly affected in both reveals 9 individual cancers (Fig 3C). This includes the 3 CTHRC1 overexpressing individual cancers (BRCA, HNSC and LIHC) identified earlier (Fig 1D and 1E). The table in Fig 3C marks cancers with significant CTHRC1 expression in purple and their significant effect on survival in pink. Multivariate survival analysis for race (data available in 7 cancers) and gender (data available in 6 cancers) was also done. In these cancers with available data, a comparison for race between, 1) White vs Not Hispanic or Latino, 2) White vs Black and 3) White vs Asian revealed the following. In HNSC, survival was affected significantly for white but not black patients. In LIHC and STAD survival was significantly affected in Asian patients but not in white patients. In BRCA, black and white patients were both significantly affected though their hazard ratios were distinctly different (Black– 5.77 and White– 1.97) (Fig 3D). This suggests black patients with “high” CTHRC1 expression (top 75 percentile) are 3.8 times more likely to have poor survival compared to white patients in the top 75 percentile. Data for gender when compared revealed a significant effect on survival in only males in BLCA and LIHC and only females in SARC. Males and females were significantly affected in HNSC, KIRC and STAD (Fig 3E). Taken together, the multivariate analysis across CTHRC1 overexpressing cancers does not reveal any distinctly conserved effect on survival across race or gender. These effects when seen do seem to be limited to individual cancer types.

### Identifying possible genes involved in CTHRC1 dependent cancers

**Fig 4:**
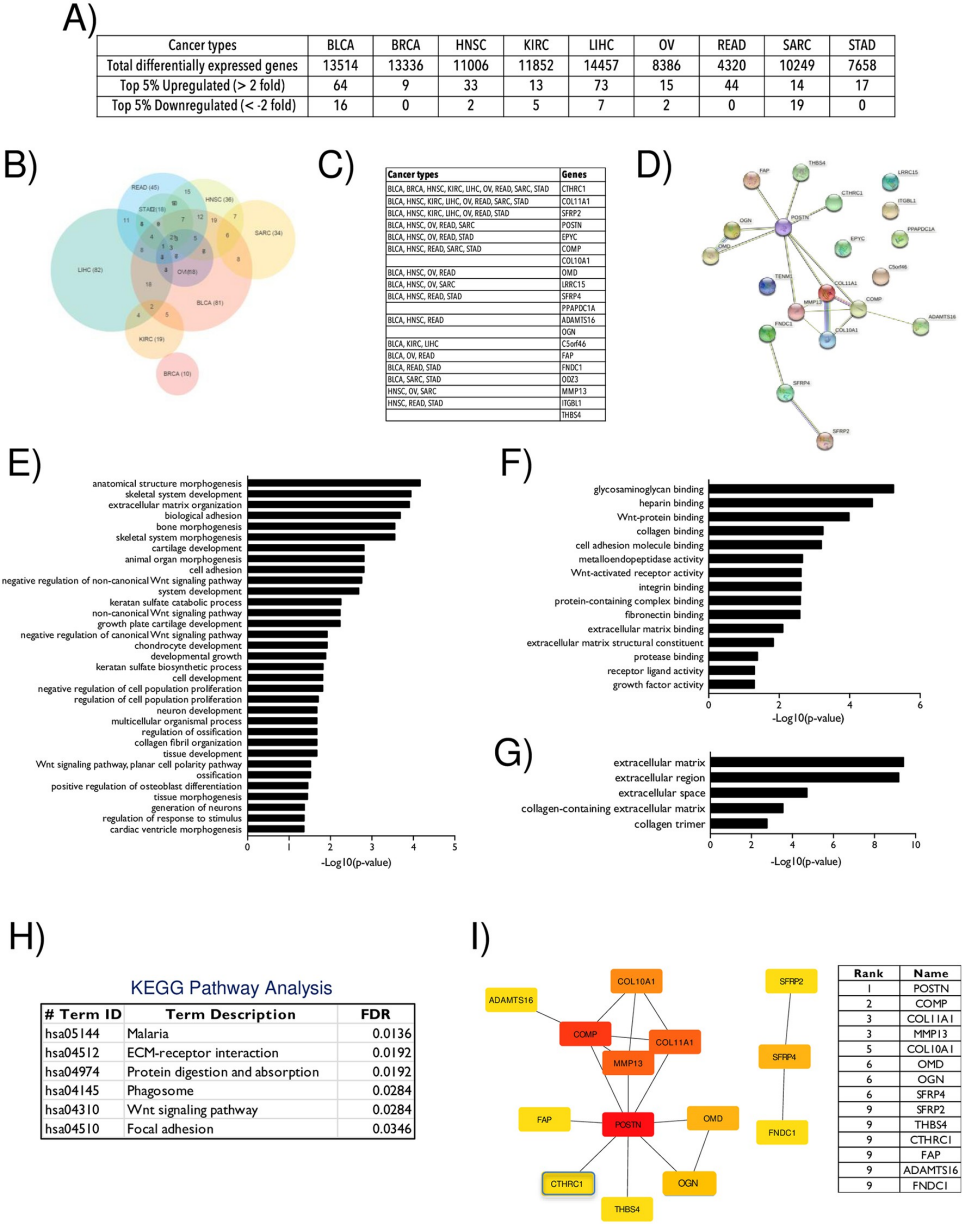
Differential gene expression analysis of CTHRC1. **A)** The table lists in the 9 selected cancers (BLCA, BRCA, HNSC, KIRC, LIHC, OV, READ, SARC and STAD–as detailed in Methods) the number of differentially expressed genes and the top 5% genes upregulated or downregulated with a 2 fold change (as detailed in Methods). **B)** This Venn diagram shows the overlap (if any) of the top 5% upregulated genes in the above listed 9 cancers. **C)** Table lists the 19 overlapping genes between 3 or more cancer types. **D)** Protein-protein interaction network constructed for CTHRC1 and its 19 differentially expressed genes using the STRING database. BLUE line marks predicted interactions from gene co-occurrence data, GREEN line marks predicted interactions based on gene neighbourhood evidence, PURPLE line marks experimentally determined known interactions, YELLOW line marks interactions based on text mining and the LIGHT BLUE line marks interactions based on database evidence. **E-F)** Functional enrichment for significant (p<0.05) **(E)** biological processes, **(F)** molecular functions and **(G)** cellular components in the STRING network analysis are listed in their descending order of significance. (**H**) The table lists the pathways identified by KEGG analysis for the STRING network in the descending order of their significance (FDR). **I)** Network of 13 hub genes identified using CytoHubba plugin in Cytoscape software. Colours of the hub genes are based on their rank which is also listed as a table (High to low).

Differential gene expression (DEG) analysis of the 9 CTHRC1 dependent cancers, BLCA, BRCA, HNSC, KIRC, LIHC, OV, READ, SARC and STAD was done to identify genes that could be regulated by CTHRC1. The top 5% of these genes with a 2 fold increase or decrease (p<0.05) in expression in each of the above cancer types were selected for further analysis (Fig 4A, S1A - S1J Files in S1 File). This identified 19 genes that were upregulated in 3 or more cancers (Fig 4B and 4C), but none that were downregulated. The Venn diagram (Fig 4B) shows the overlapping upregulated genes. The 19 upregulated genes include Collagen type XI alpha 1 chain (COL11A1), Secreted Frizzled related protein 2 (SFRP2), Periostin (POSTN), Epiphycan (EPYC), Cartilage oligomeric matrix protein (COMP), Collagen type X alpha 1 chain (COL10A1), Osteomodulin (OMD), Leucine rich repeat containing 15 (LRRC15), Secreted frizzled related protein 4 (SFRP4), Phosphatidic acid phosphatase type 1A (PPAPDC1A), ADAM metallopeptidase with thrombospondin type 1 motif (ADAMTS16), Osteoglycin (OGN), Chromosome 5 open reading frame 46 (C5orf46), Fibroblast activation protein alpha (FAP), Fibronectin type III domain containing 1 (FNDC1), Teneurin transmembrane protein 1 (ODZ3/TENM1), Matrix metalloproteinase 13 (MMP13), Integrin subunit beta like 1 (ITGBL1) and Thrombospondin 4 (THBS4) (Fig 4C). They were used to construct a protein-protein interaction network using the STRING database (Fig 4D). Co-expression, database information and text mining are what largely contribute to the making of this network. There is hence a vital need to experimentally validate this network. Functional enrichment analysis of this network revealed ECM organization and adhesion to be among the top 5 biological processes regulated by these network genes (Fig 4E). At the molecular level, these proteins bind Fibronectin, Collagen, cell adhesion molecules and integrins among others (Fig 4F). Proteins in this network almost exclusively belong to the extracellular compartment (extracellular matrix, region and space) (Fig 4G). KEGG analysis of this network further shows their involvement in ECM-receptor interaction, Wnt and focal adhesion signalling (Fig 4H). cytoHubba tool identified 14 hub genes (with a score ≥1) from this predicted network which includes, POSTN, COMP, COL11A1, MMP13, COL10A1, OMD, OGN, SFRP4, SFRP2, THBS4, CTHRC1, FAP, ADAMTS16 and FNDC1 (Fig 4I). Of these POSTN ranks highest and has the most number of connections, which also includes CTHRC1.

**Fig 5:**
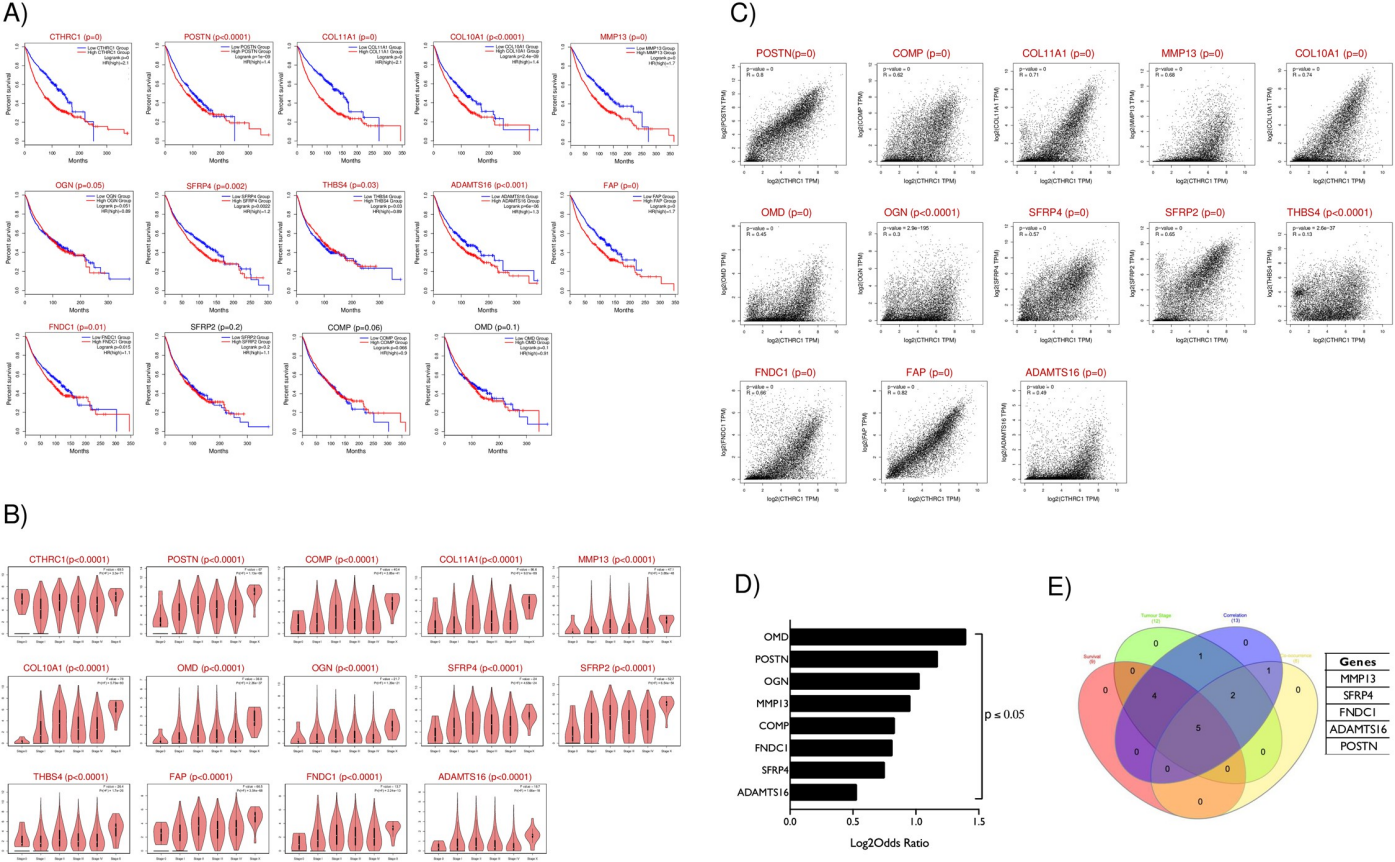
Validation of CTHRC1 and its hub genes in cancers. **A)** Graph represents percentage survival in 30 cancers with “high” (RED plot) vs “low” (BLUE plot) expression for CTHRC1 or each of its 13 hub genes (POSTN, COMP, COL11A1, MMP13, COL10A1, OMD, OGN, SFRP4, SFRP2, THBS2, FAP, FNDC1 and ADAMTS16) using GEPIA2 database. The significance of the difference in survival is listed above each graph. p values are as indicated above the graph. Genes with significance (p ≤ 0.05) are listed in RED and those lacking significance in BLACK. p values = 0 are representative of very high significance. **B)** Violin plot shows the expression of CTHRC1 and each of its 13 hub genes across pathological stages in 30 cancers analyzed using the GEPIA2 database. Differences across the stages of cancer for each gene of interest was calculated using the ANOVA test and significance was reported. p values are as indicated above the graph. Genes with significance (p ≤ 0.05) are listed in RED. **C)** Scatter plots show the Spearman correlation analysis for CTHRC1 and its 13 hub genes in 30 cancers using GEPIA2. p values are as indicated above the graph. Genes with significance (p ≤ 0.05 or p = 0) are listed in RED. **D)** Bar graph shows log2 odds ratio from the cBioPortal for statistically significant co-occurrence between CTHRC1 and hub genes of interest (8 genes) in 30 cancer types. **E)** This Venn diagram shows the overlap of genes that significantly affects survival and tumour staging and are related in correlation and co-occurrence analysis in 30 cancer types. The table lists the 5 overlapping genes detected in this analysis.

To validate the significance of these hub genes with CTHRC1, we first analyzed the effect on pan-cancer survival of CTHRC1 and these 13 hub genes using the TCGA data through the GEPIA2 portal. Like CTHRC1, COL11A1, MMP13, COL10A1, POSTN, OGN, SFRP4, FAP, ADAMTS16 and FNDC1 were all seen to significantly affect survival in cancers with “high” expression (top 75 percentile) relative to “low” (bottom 25 percentile) (Fig 5A). THBS4 however significantly affects survival in cancers with “low” (bottom 25 percentile) relative to “high” (top 75 percentile) (Fig 5A). We used data from the GEPIA2 portal to determine if the change in expression of CTHRC1 and 13 hub genes is associated with tumour grade. Statistical analysis of this change across tumour grades by ANOVA showed all hub genes and CTHRC1 expression to indeed be tumour stage-dependent (Fig 5B). Spearman correlation analysis showed a positive correlation between all the 13 hub genes and CTHRC1 (Fig 5C). cBiportal analysis also showed a significant co-occurrence between CTHRC1 and OMD, POSTN, OGN, MMP13, COMP, SFRP4, ADAMTS16 and FNDC1 (Fig 5D). Based on the significant effect they have on survival, tumour grade, correlation and co-occurrence analysis, we identified five genes, POSTN, MMP13, SFRP4, ADAMTS16 and FNDC1 as the most likely mediators of CTHRC1 dependent function in cancers (Fig 5E–represented in the Venn diagram).

**Fig 6:**
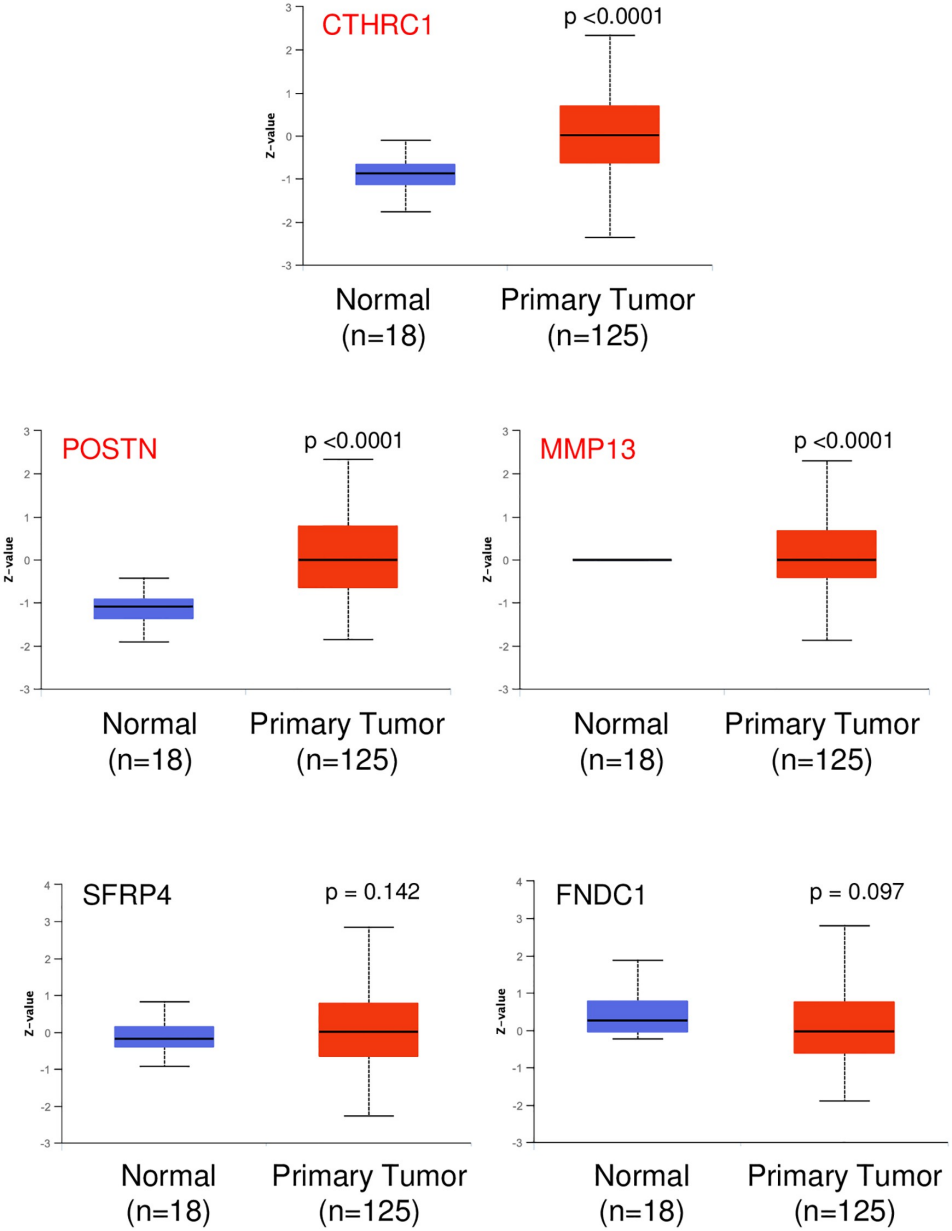
Protein levels of CTHRC1 and network genes in breast cancer. Graphs represent protein levels of CTHRC1 and shortlisted network genes (POSTN, MMP13, SFRP4 and FNDC1) in normal (BLUE) versus tumour tissue (RED) data from the UALCAN Portal. The box plot shows the median ± standard deviation. p values are as indicated and calculated using the students t-test. Genes with significance (p <0.05) are listed in RED and those lacking significance in BLACK.

It would hence be of interest to look at the protein expression data in cancers for CTHRC1 and the now identified genes of interest, POSTN, MMP13, SFRP4, ADAMTS16 and FNDC1. Of the 9 cancers shortlisted in the pan-cancer analysis based on CTHRC1 overexpression and its effect on cancer survival (Fig 3A–3C), BRCA, HNSC and LIHC were also shortlisted in the individual cancer study based on their CTHRC1 expression and its effect on survival. Protein expression data for most of the network genes (4 out of 5) of interest was available for only BRCA (breast cancer) in the UACLAN portal. Mass spectrometric data from 125 breast tumour samples showed CTHRC1, POSTN and MMP13 to all be significantly higher in breast tumour tissue relative to normal while SFRP4 and FNDC1 levels were unaffected (Fig 6A).

**Fig 7:**
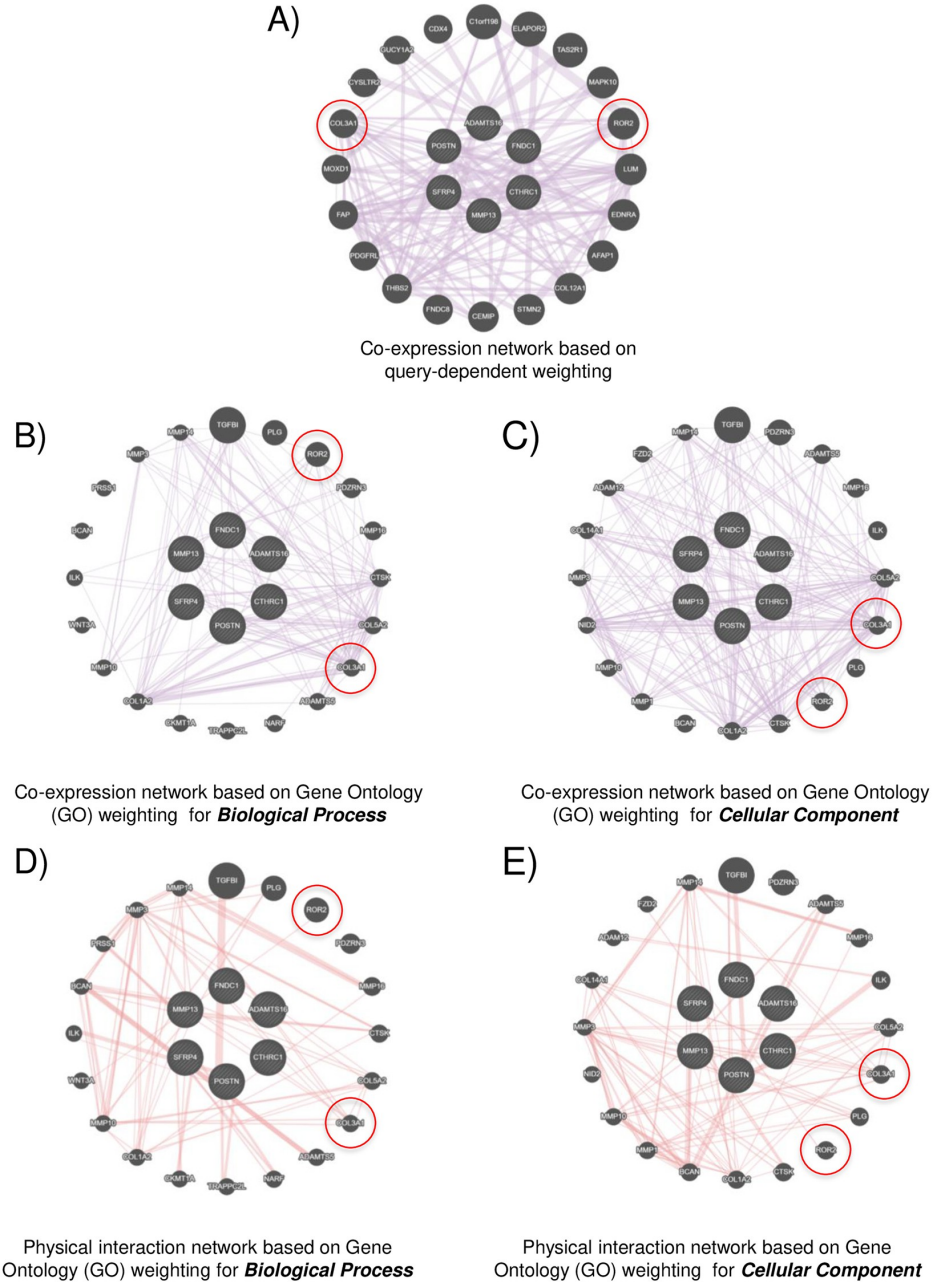
Functional Network analysis of CTHRC1 and its 5 hub genes. Network analysis of CTHRC1 and its 5 hub genes (POSTN, MMP13, FNDC1, SFRP4 and ADAMTS16) identifies COL3A1 and ROR2 genes in both co-expression and physical interaction categories. **A)** Image shows co-expression network based on query genes, **B-C**) Images shows co-expression network based on **(B)** biological processes and (C) cellular component. **D-E)** Image shows physical interaction network based on **(D)** biological processes and **(E)** cellular component.

**Fig 8:**
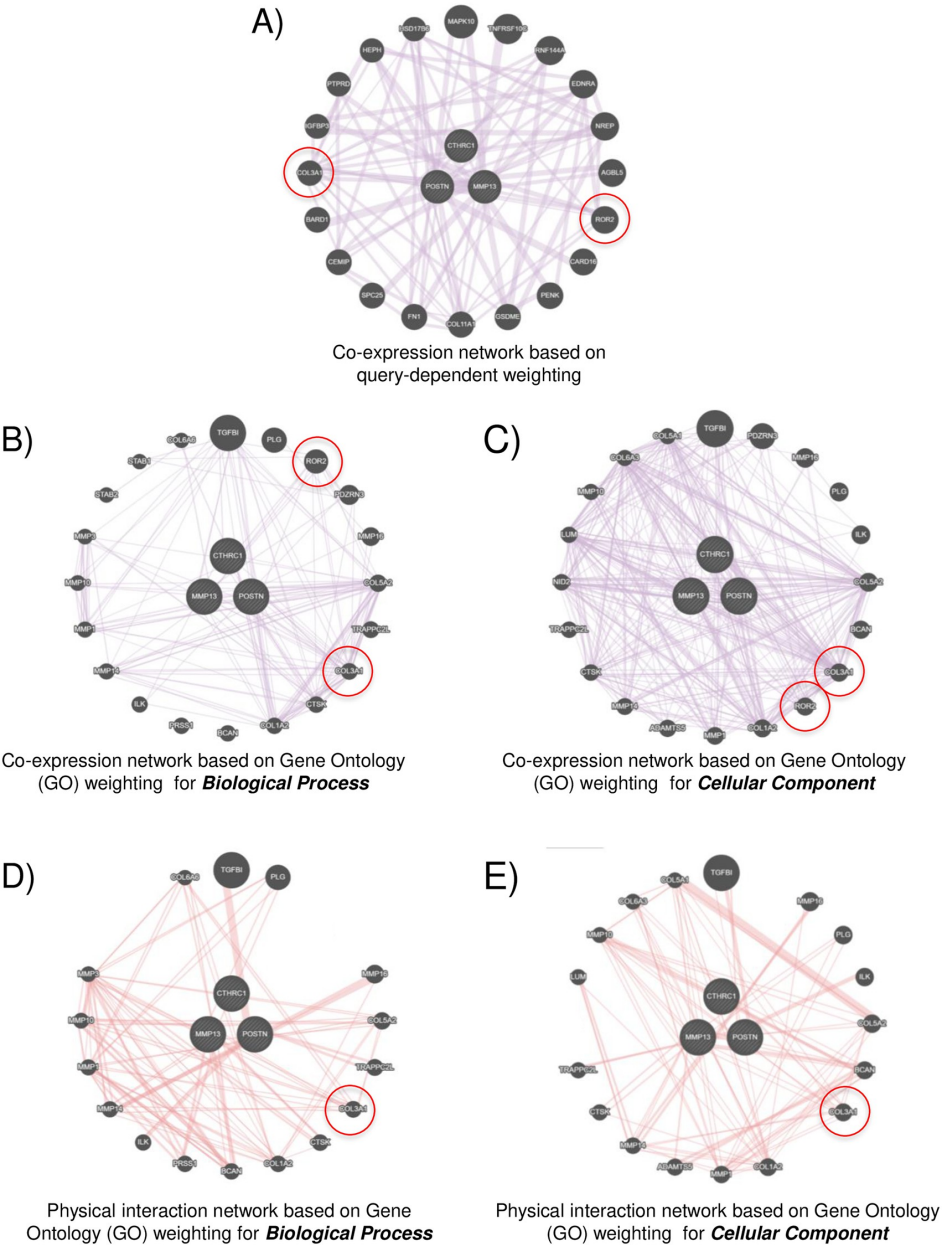
Functional Network analysis of CTHRC1, POSTN and MMP13. Network analysis of CTHRC1, POSTN and MMP13 identifies COL3A1 in both co-expression and physical interaction categories. **A)** Image shows co-expression network based on query genes, **B-C**) Images shows co-expression network based on **(B)** biological processes and **(C)** cellular component. (**D-E)** Image shows physical interaction network based on **(D)** biological processes and **(E)** cellular component.

To further test if CTHRC1 and its related hub genes (POSTN, MMP13, SFRP4, ADAMTS16 and FNDC1) share functional networks, we performed network analysis using GeneMANIA online database [34] and compared their co-expression and physical interaction networks. The co-expression network based on query-dependent weighting (Fig 7A) is seen to be rich in core matrisome genes, cell receptors and intracellular signalling molecules (S1A Fig). Co-expression and physical interaction networks based on Gene Ontology (GO) weighting for biological process and cellular components were also evaluated (Fig 7B–7E). These networks were enriched in core matrisome genes and proteases (S1B–S1E Fig). Query and Gene Ontology-based networks when compared identified Collagen type III alpha chain 1 (COL3A1) and receptor tyrosine kinase like receptor 2 (ROR2) as two genes that were conserved among these networks and could hence be involved in regulating CTHRC1 and its hub gene-mediated regulation of the tumour matrisome (Fig 7A–7E –labelled with the red circle). In breast cancer (BRCA) CTHRC1 is overexpressed with hub genes POSTN and MMP13 (Fig 6), leading us to do evaluate their co-expression network based on query-dependent weighting and co-expression and physical interaction network based on Gene Ontology (GO) weighting for biological process and cellular components. These co-expression networks when compared confirmed COL3A1 and ROR2 to be genes conserved (Fig 8A–8C- labelled with the red circle), as seen earlier (Fig 7). Physical interaction networks however detected only COL3A1 (Fig 8D and 8E), suggesting it could be a gene of interest that CTHRC1, POSTN and MMP13 could use to regulate the tumour matrisome in breast cancers. Together they further emphasize the possible role overexpression of these matrisome genes and their crosstalk as part of a regulatory and/or functional network could have in driving the impact of the ECM through CTHRC1 in cancers.

## Discussion

In the past decade, the ECM has emerged as a key player in the progression, diagnosis, and treatment of cancers. Changes in the ECM composition and structure have been known to promote migration and invasion of cancer cells [2, 22, 35, 36]. Altered ECM deposition is further associated with poor prognosis in multiple cancers [2, 3]. Recent studies have shown that CTHRC1 is overexpressed in cancers and associated with poor prognosis [37]. These studies have specifically implicated CTHRC1 with immune infiltration in Kidney and Brain cancers. *Peng at al* [38] reported that in addition to being upregulated in high-grade gliomas, CTHRC1 expression correlated with genes associated with the Wnt Signaling pathway (DVL3, DVL1, DVL2, ROR2, WNT3A, FZD6 and FZD5). Our study in evaluating 1027 matrisome genes across cancers, has identified a novel set of genes (MMP13, FNDC1, SFRP4 and ADAMTS16) that could work with CTHRC1 to regulate cancer progression.

Using TCGA data from 30 cancer types, we evaluated 1027 matrisome genes stringently analyzing their copy number, expression data with their effect on cancer survival and identified the top overexpressed (n = 23) and downregulated (n = 17) genes of interest across cancers. We further used a similar criterion to evaluate matrisome genes in individual cancers and identify genes affecting survival in 2 or more cancers. The intent here was to compare the pan- and individual cancer analysis to identify matrisome gene(s) that consistently show differential expression and affect cancer survival. This while eliminating possible false positives will also ensure that the genes eventually selected are strong candidates for a pan-cancer role.

This identified 3 matrisome genes, CTHRC1, PDGFA and IL7 to be prominent candidates of which only CTHRC1 was upregulated in pan-cancer and 3 individual cancers making it the matrisome gene of interest (Figs 1D and 2F, ***table in red***). The expression and effect on survival of CTHRC1 were hence compared across 30 cancer types identifying 9 cancers where both were affected (BLCA, BRCA, HNSC, KIRC, LIHC, OV, READ, SARC and STAD). Differential gene expression analysis of these cancers to identify genes with a 2-fold change (increase/decrease) in 3 or more cancers led us to 19 matrisome genes that could work with CTHRC1. STRING analysis of these 19 genes further identified 13 hub genes. This network data is largely based on predictions from text mining and suggests CTHRC1 to primarily talk to POSTN which in turn communicates with other hub genes. Could the CTHRC1-POSTN connect be of significance to their role as part of the matrisome in cancers remains to be tested experimentally. Additional hub genes MMP13, SFRP4, ADAMTS16 and FNDC1 also significantly affect survival with their expression across tumour grades comparable to CTHRC1. They also show a significant correlation and co-occurrence with CTHRC1 across cancers. Proteomics data for breast cancers in revealing CTHRC1 overexpression with POSTN and MMP13 further strengthens the need to look at this gene network in other cancers.

**CTHRC1** is a known regulator of collagen synthesis [39], shown to inhibit collagen type I and III transcripts [40], with KO mice showing reduced type I collagen levels [41]. Fibroblasts from CTHRC1 KO mice show significant downregulation of genes involved in ECM organization and collagen biosynthesis [42]. CTHRC1 is also known to regulate Wnt signalling and enhances the binding of Wnt3A with the Frizzled receptors [43]. Both POSTN and MMP13 are known to independently regulate the collagen [44, 45]. **POSTN** plays an important role in ECM structure and organization via its interaction with BMP1 to accelerate collagen cross-linking [12]. POSTN null mice exhibit aberrant collagen fibrillogenesis in the periosteum and a decrease in collagen cross-linking in the skin, tendons, and heart [44]. CTHRC1 and POSTN along with collagen are both highly expressed by the same cluster of cells in fibrotic lungs and hearts [42, 46]. **MMP13** binds collagen through its c terminal domain and cleaves collagen [45]. MMP13 KO mice implanted with mammy tumour cells show increased lung metastasis as a result of increased collagen synthesis and altered collagen structure and organization [47]. Similarly, **SFRP4** (a known Wnt antagonist) could also regulate collagen structure and organization by controlling Wnt signalling via beta-catenin [48]. Exogenous addition of SFRP4 decreases scar formation by regulating ECM deposition in infarcted hearts of mice [49].

Studies using cardiac fibroblasts have revealed the presence of crosstalk between TGFß and Wnt signalling to regulate the fibrotic response [50]. CTHRC1 binds TGFß and promotes its activation in colorectal cancer cells [51]. It also interacts with TGFß receptor II and TGFß receptor III to stabilize the ligand-receptor pathway and promote liver metastasis [51]. Hence, CTHRC1 could regulate collagen synthesis and organization by modulating both TGFß and Wnt signalling pathways. Increased activation of TGFß induces POSTN and CTHRC1 expression [52]. Like CTHRC1, **ADAMTS16** also physically binds TGFß to promote its activation [53] and loss of ADAMTS16 in rats results in a reduced TGFß activation [39]. In a mice model for heart failure, ADAMTS16 is upregulated, its expression correlating with collagen expression [53]. This TGFß -ADAMTS16 feedback could regulate collagen synthesis, organization and degradation.

Similar to collagen, these CTHRC1 related network genes could also act to regulate fibronectin synthesis, organization and degradation. FN and collagen organization are interdependent [54, 55]. Cancer-associated fibroblasts secrete high levels of FN to form a highly organized FN rich ECM which promotes cancer cell migration and invasion [56, 57]. Such a fibronectin matrix promotes cancer cell survival during dormancy while its MMP mediated degradation promotes proliferation [58]. **CTHRC1** and FN are both overexpressed in melanomas where they are found to be localized in similar regions [59]. **POSTN** which binds FN also promotes its synthesis via the JNK pathway [60]. **MMP13** cleaves FN which also upregulates its expression using the same pathway [61]. **ADAMTS16** also cleaves FN inducing MMP3 expression to promote FN degradation [62]. Exogenous Wnt ligands can promote FN synthesis [63] that **SFRP4** dependent regulation of Wnt signalling [49, 64] could also regulate. During wound healing, CTHRC1, MMP13, ADAMTS16, SFRP4, POSTN and FNDC1 are all significantly upregulated by myofibroblasts to regulate ECM deposition [65]. POSTN binding to FN and collagen [66] could further regulate the ECM to drive tumour cell migration and invasion.

In breast cancer, CTHRC1 gene and protein expression is upregulated with MMP13 and POSTN. The UALCAN proteomic data while currently limited to 125 breast cancer samples (of the total TCGA cohort of 1080), does support the same. Though CTHRC1 and POSTN upregulation in breast cancer is associated with poor prognosis [67], no direct interaction between them is reported. In breast cancer, tumour-associated collagen signatures are of prognostic significance. Aligned collagen bundles are negatively correlated with breast cancer survival [68]. CTHRC1 with POSTN and MMP13 could potentially affect ECM remodelling which in turn could aid in tumor cell migration and invasion [69, 70]. CTHRC1 secreted by cells could also independently bind collagen and regulate its assembly and organization. This could also modulate how secreted growth factors (i.e.,TGFb and Wnt) are sequestered by the matrix in breast cancer cells [71]. CTHRC1 expression is associated with metastasis to the bone [72]. In breast cancer patients with high periostin, the risk of bone metastases is enhanced by elevated CTHRC1 expression [68]. This when considered with the known role for MMP13 in bone metastasis of breast cancer [73, 74] strongly supports a role for CTHRC1-POSTN-MMP13 crosstalk in mediating the same. Understanding how this crosstalk facilitates bone metastasis remains a vital open question.

Further experimental validation of the predictive bioinformatics data will be vital to establish the regulatory and/or functional crosstalk between CTHRC1 and POSTN, MMP13, SFRP4, FNDC1 and ADAMTS16. CTHRC1 is susceptible to proteolysis and cleaved CTHRC1 has been reported to be a better inhibitor of collagen synthesis [40]. If MMP13 or ADAMTS16 could regulate CTHRC1 cleavage remains to be tested. CTHRC1, ADAMTS16 and POSTN are all reported to bind TGFß [12, 39] which could as a point of convergence for their regulatory crosstalk. CTHRC1 and POSTN expression [53] are directly induced upon TGFß activation which could be one of the key players in influencing the CTHRC1 related matrisome network. Whether FNDC1, SFRP4, ADAMTS16 and MMP13 are regulated by CTHRC1 independent of TGFß remains to be evaluated.

Could the CTHRC1 network also regulate other growth factors (Wnt, EGF, FGF etc) remains to be verified. A joint role for these proteins in ECM remodelling (through collagen and fibronectin) could regulate tumour cell migration and invasion [69, 70]. The cross-linking of collagen fibers controls their density and packing order which could regulate ECM stiffness to further drive cancer progression [75–80] downstream of CTHRC1.

Our functional network analysis of CTHRC1 and its hub genes using GeneMANIA has identified COL3A1 and ROR2 to be a part of the co-expression and physical interaction networks. CTHRC1 has been shown to regulate type III collagen synthesis and binds ROR2 to regulate planar cell polarity during development [81]. Changes in ECM composition and remodelling drive tumour progression [82]. Collagen fibers are seen to be less dense, shorter, straighter, thinner, and more aligned with one another in breast cancer [83] which can predict their pathology and outcomes [84]. Both COL3A1 and ROR2 have been implicated in breast cancer pathogenesis [85–88]. ROR2 has been shown to act as an oncogene to promote breast cancer progression. COL3A1 is highly expressed by the tumour stroma and associated with increased survival in breast cancer patients [89]. Secreted COL3A1 causes a wavy collagen fiber orientation promoting tumour dormancy in breast cancer [90] possibly through a DDR pathway (Discoidin domain receptor—tyrosine kinase proteins activated by collagen) to limit metastasis [88, 90]. COL3A1 expression could have implications as a vital biomarker in breast cancer [88]. In Esophageal Cancer COL3A1 is overexpressed with POSTN [91] which further emphasizes the joint role they could have with CTHRC1. Thus, in identifying CTHRC1 and the network of genes it works with across cancers, this study not only helps reveal the possible role POSTN, MMP13, SFRP4, FNDC1 and ADAMTS16 could have in regulating the impact of the matrisome in cancers but also highlights the role such a network could have in sustaining the same.

## Supporting information

S1 Fig Protein categories of the functional network analysis of CTHRC1 and its 5 hub genes. Nested bar graphs represent the percentage of proteins that belong to each category in **(A)** the network analysis based on query genes, (B) co-expression network based on biological processes or (C) cellular component and physical interaction network based on **(D)** biological processes or **(E)** cellular component. Each colour represents a distinct protein subcategory.
(PDF)

S1 File **S1A is the summary file of the top 5% genes for 9 cancers. S1B - S1J** are the raw data output files of the differential gene expression analysis of the 9 cancers (Fig 4A) where CTHRC1 is upregulated and affects survival.
(ZIP)
