# Supplementary material for: A pan-cancer analysis of matrisome proteins reveals CTHRC1 and a related network as major ECM regulators across cancers
Source: PLoS One. 2022 Oct 3;17(10):e0270063. doi: 10.1371/journal.pone.0270063 (PMC9529084; doi:10.1371/journal.pone.0270063)
Supplement: S1 Fig — Nested bar graphs represent the percentage of proteins that belong to each category in (A) the network analysis based on query genes, (B) co-expression network based on biological processes or (C) cellular component and physical interaction network based on (D) biological processes or (E) cellular component. Each colour represents a distinct protein subcategory. (PDF) [file pone.0270063.s001.pdf]

*Harikrishnan et al., Supplementary Figure 1*

A)

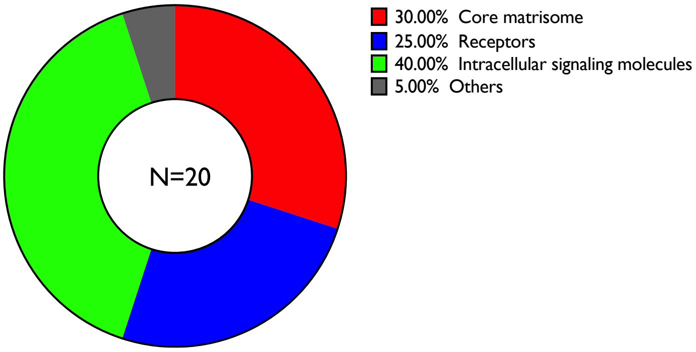


Co-expression network based on query-dependent weighting

B)

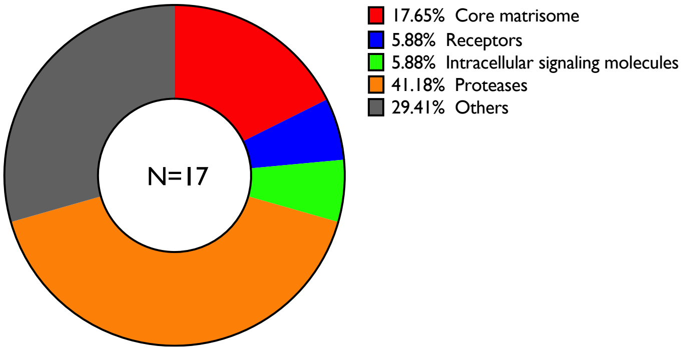


Co-expression network based on Gene Ontology (GO) weighting for ***Biological Process***

C)

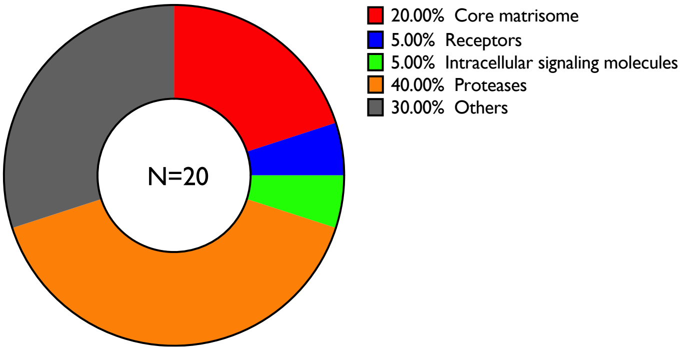


Co-expression network based on Gene Ontology (GO) weighting for ***Cellular Component***

D)

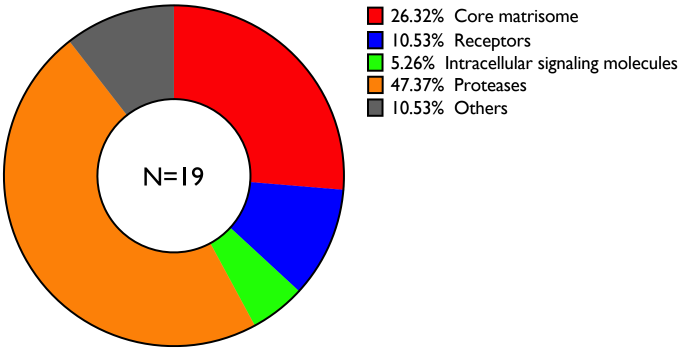


Physical interaction network based on Gene Ontology (GO) weighting for ***Biological Process***

E)

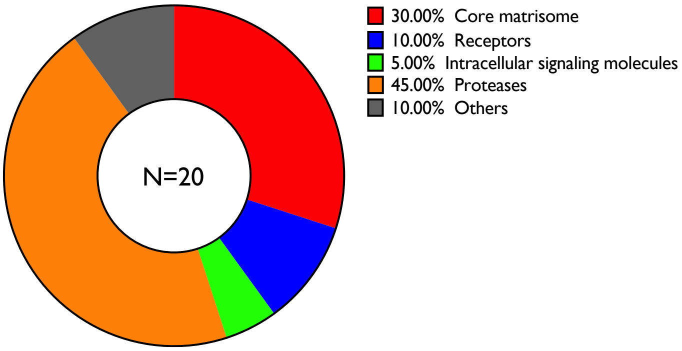


Physical interaction network based on Gene Ontology (GO) weighting for ***Cellular Component***
